# Effect of serial pig passages on the adaptation of an avian H9N2 influenza virus to swine

**DOI:** 10.1371/journal.pone.0175267

**Published:** 2017-04-06

**Authors:** Jose Carlos Mancera Gracia, Silvie Van den Hoecke, Xavier Saelens, Kristien Van Reeth

**Affiliations:** 1 Laboratory of Virology, Department of Virology, Parasitology and Immunology, Faculty of Veterinary Medicine, Ghent University, Merelbeke, Belgium; 2 Center for Medical Biotechnology, VIB, Ghent, Belgium; 3 Department of Biomedical Molecular Biology, Ghent University, Ghent, Belgium; University of Geneva, SWITZERLAND

## Abstract

H9N2 avian influenza viruses are endemic in poultry in Asia and the Middle East. These viruses sporadically cause dead-end infections in pigs and humans raising concerns about their potential to adapt to mammals or reassort with human or swine influenza viruses. We performed ten serial passages with an avian H9N2 virus (A/quail/Hong Kong/G1/1997) in influenza naïve pigs to assess the potential of this virus to adapt to swine. Virus replication in the entire respiratory tract and nasal virus excretion were examined after each passage and we deep sequenced viral genomic RNA of the parental and passage four H9N2 virus isolated from the nasal mucosa and lung. The parental H9N2 virus caused a productive infection in pigs with a predominant tropism for the nasal mucosa, whereas only 50% lung samples were virus-positive. In contrast, inoculation of pigs with passage four virus resulted in viral replication in the entire respiratory tract. Subsequent passages were associated with reduced virus replication in the lungs and infectious virus was no longer detectable in the upper and lower respiratory tract of inoculated pigs at passage ten. The broader tissue tropism after four passages was associated with an amino acid residue substitution at position 225, within the receptor-binding site of the hemagglutinin. We also compared the parental H9N2, passage four H9N2 and the 2009 pandemic H1N1 (pH1N1) virus in a direct contact transmission experiment. Whereas only one out of six contact pigs showed nasal virus excretion of the wild-type H9N2 for more than four days, all six contact animals shed the passage four H9N2 virus. Nevertheless, the amount of excreted virus was significantly lower when compared to that of the pH1N1, which readily transmitted and replicated in all six contact animals. Our data demonstrate that serial passaging of H9N2 virus in pigs enhances its replication and transmissibility. However, full adaptation of an avian H9N2 virus to pigs likely requires an extensive set of mutations.

## Introduction

Avian influenza viruses (AIVs) of the H9N2 subtype emerged from the natural waterfowl reservoir and have become endemic in poultry in Asia and the Middle East since the early 1990s [1]. Genetic characterization and phylogenetic analysis revealed that most poultry H9N2 viruses belong to one of three different lineages: G1-like, Y280-like and Y439-like, also known as Korean-like [2, 3]. In 1999, H9N2 viruses were for the first time isolated from two patients with mild respiratory symptoms [4]. These human isolates were genetically and antigenically similar to the G1-like lineage [5]. Later, from 2002 until 2016, H9N2 viruses belonging to the G1-like and Y280-like lineages have been occasionally reported in humans [4, 6, 7]. The first H9N2 virus that was isolated from swine belonged to the Y280-like lineage and was reported in Hong Kong in 1998 [8]. Since then, different lineages of H9N2 viruses were sporadically detected in swine in Mainland China [9–11]. Although these infections of humans and pigs indicate that H9N2 viruses can overcome the species barrier without prior adaptation, no human-to-human or pig-to-pig transmission has been reported so far [12].

Influenza A virus infection is mediated via the attachment of the viral hemagglutinin (HA) receptor-binding site (RBS) to sialyloligosaccharides present on the host cell surface. Avian influenza viruses preferentially bind sialic acids linked to galactose by an α2,3 linkage (Siaα2,3Gal). Conversely, most human and swine influenza viruses more readily bind to receptors that contain terminal α2,6-linked sialic acid (Siaα2,6Gal) [13]. Because of the predominant expression of Siaα2,6Gal in the human and swine upper respiratory tract [14], a switch from Siaα2,3Gal to Siaα2,6Gal receptor binding preference is considered an important factor for avian influenza virus adaptation to mammals [15]. Specific amino acid substitutions in the HA RBS have been associated with enhanced Siaα2,6Gal binding [16]. For H1 subtype viruses, for example, a crucial role has been assigned to the substitution of glutamic acid by aspartic acid at position 190 (HA-E190D) (H3 numbering), and glycine by aspartic acid at position 225 (HA-G225D). For H2 and H3 subtypes, the crucial changes were substitution of glutamine by leucine at position 226 (HA-Q226L) and glycine by serine at position 228 (HA-G228S) [17–19]. A significant proportion of poultry H9N2 field isolates contain the HA-Q226L mutation, which has been shown to enhance binding to Siaα2-6Gal and replication of these viruses in human airway epithelial cells *in vitro* [20, 21]. In addition, Wan *et al*. reported that HA-Q226L increased virus replication and direct contact transmission of H9N2 in ferrets, which are considered the “gold standard” model for human influenza pathogenesis and transmissibility studies [22, 23]. However, virus transmission of these H9N2 viruses was still less efficient compared to that of human adapted viruses [23]. This raises the critical question: which other changes are required to make avian H9N2 viruses fully adapted to a mammalian host?

Pigs are important natural hosts for influenza A viruses. These animals have a similar sialic acid receptor distribution as humans [14], and they are susceptible to both avian and human influenza viruses [24, 25]. Since 1985, pigs have been proposed as intermediate hosts for the adaptation and transmission of avian influenza viruses from birds to humans [26, 27]. However, the exact mechanisms by which AIVs may fully adapt to pigs and cause a pandemic are not completely understood. Previous studies reported on the pathogenicity, infectivity and transmissibility of different H9N2 viruses in swine based on different experimental approaches [24, 25, 28–30]. Furthermore, the infection was subclinical in all cases. Intranasal inoculation of pigs with H9N2 viruses isolated from humans or chickens since the late 1990s resulted in nasal virus excretion for more than one day, but transmission was either undetectable or far less efficient than that of endemic swine influenza viruses [28–30]. Serial passaging of AIVs in mammals is a proven strategy to promote the selection of virus variants that are better adapted to replicate in, and transmit between this non-natural host [31–33]. Here we sought to improve the adaptation of a wild-type H9N2 AIV by performing ten blind serial passages in influenza naïve pigs. To mimic the natural situation as much as possible, we used virus isolated from the nasal mucosa as inoculum for every subsequent passage in an attempt to select for viruses with improved nasal virus shedding and transmission. By doing so, we obtained an H9N2 virus with a predominant mutation at position 225 in HA that replicated and transmitted better in pigs than the parental virus.

## Materials and methods

### Ethics statement

The experiments were authorized and supervised by the Ethical and Animal Welfare Committee of the Faculty of Veterinary Medicine of Ghent University, with the EC2013/63 number.

### Influenza viruses

A/quail/Hong Kong/G1/1997 (H9N2) was isolated from quail at a live bird market and underwent seven passages in eleven-day-old embryonated chicken eggs, three passages in Madin-Darby Canine Kidney cells (MDCK), and two passages in pigs. After each pig passage the virus was also passaged once in MDCK cells to grow a large stock to be used in the experiment. The virus stock used in the experiment shares at least 99% identity at the nucleotide and protein levels with the original A/quail/Hong Kong/G1/1997 virus (GenBank accession numbers: AF156435.1, AF156421.1, AF156449.1, AF156378.1, AF156407.1, AF156396.1, AF156463.1 and AF156477.2). This strain was selected because it is representative for the G1-like lineage and contains the HA-Q226L substitution that is known to enhance human-like receptor specificity.

A/California/04/2009 pandemic H1N1 (pH1N1) virus underwent three passages in MDCK cells, one in eggs and a last passage in MDCK cells before use. This virus is representative of the pH1N1 viruses that circulate worldwide in swine.

### Animals

Forty-seven three-week-old piglets were purchased from a commercial herd that is serologically negative for swine influenza and porcine reproductive and respiratory syndrome virus. Before the start of the experiments, all animals were free of influenza virus antibodies as demonstrated by a commercial blocking anti-influenza A nucleocapsid ELISA (ID-VET) and by virus neutralization (VN) tests using A/swine/Belgium/1/98 (H1N1), A/swine/Flanders/1/98 (H3N2), A/swine/Gent/7625/99 (H1N2) swine influenza viruses (SIVs), as well as A/California/04/09 (pH1N1). Upon arrival, the animals were housed in a biosafety level-2 (BSL-2) HEPA filtered isolation unit for at least two days to allow their acclimatization.

### Blind serial passages of avian H9N2 influenza virus in pigs

For the first passage, two pigs were housed in a biosafety level-3 (BSL-3) HEPA filtered isolator and inoculated intranasally with three ml of phosphate-buffered saline (PBS) containing 10^6.2^ TCID_50_ of A/quail/Hong Kong/G1/1997 (H9N2). For inoculation unanesthesized pigs were held in a vertical position with the neck stretched. The inoculum (1.5 ml per nostril) was gradually instilled into the nasal cavity by insertion of a fifteen mm canula attached to a syringe. To determine virus excretion in the nose, individual pigs were swabbed daily from 0 to four days post-inoculation (dpi). At four dpi both animals were humanely euthanized by slow injection of an overdose (≥ 100 mg/kg) of pentobarbital in the jugular vein. At the time of necropsy, the following tissue samples were collected for virus titrations: nasal mucosa respiratory part (i.e. nasal turbinates), nasal mucosa olfactory part (i.e. ethmoid labyrinth), trachea (upper and lower half) and five different samples representative of the entire lung. The nasal mucosae (respiratory part) were pooled and a 20% (w/v) tissue homogenate was prepared and used to inoculate two pigs with this passage one virus inoculum. Ten blind (meaning that the viral load in the inoculum was not known prior to the infection) serial passages were performed in this way. Nasal swabs from all pigs, tissue samples and the inocula used for the subsequent passages were titrated in MDCK cells.

### Swine transmission studies

The inoculum giving the highest virus isolation rates and virus titers throughout the respiratory tract and in nasal swabs was examined for its transmission between pigs. We compared the transmissibility of mentioned virus to that of the parental H9N2 and that of the pH1N1. Nine three-week-old pigs were used. At 0 dpi (= 2 days before the start of contact transmission), three pigs were housed in three separate BSL-3 HEPA filtered isolators (one index pig per isolator) and intranasally inoculated with 10^6.5^ TCID_50_ of the respective virus. Forty-eight hours pi, two pigs were introduced in each isolator allowing direct contact with the inoculated pig. All animals were clinically monitored and nasal swabs for virus isolation were collected daily from all pigs during eleven days after cohousing. Sixteen dpi, the animals were relocated to a BSL-2 HEPA filtered isolation unit. Serum samples were collected at 0, 16, 23 and 30 dpi and 0, 14, 21 and 28 days post-contact (dpc).

### Growth kinetics of parental and passaged virus

To compare the single-step growth curves of the parental, passaged virus and pH1N1 confluent monolayers of MDCK cells were inoculated with an identical multiplicity of infection of 5 MOI per cell at 37°C. After one hour of incubation the cells were washed with phosphate-buffered saline (PBS) containing 10 IU/ml penicillin and 10 μg/ml streptomycin to remove unbound virus particles and overlaid with Minimal Essential Medium (MEM) containing supplements (1 mg/ml lactalbumin, 100 IU/ml penicillin, 10 μg/ml streptomycin, 50 μg/ml gentamycin and 2 μg/ml trypsin). The three viruses were tested in triplicate, supernatants were collected at 0, 4, 6, 12 and 24 hours post inoculation and titrated in MDCK cells as described below.

### Virus titrations

Cotton swabs were weighed before and after collection to determine virus titers per 100 milligram nasal secretions. Nasal swab samples from both nostrils were suspended in 1 ml PBS supplemented with 10% fetal bovine serum, 100 IU/ml penicillin and 100 μg/ml streptomycin and mixed vigorously at 4°C for 1 hour. Tissue samples were weighed and grounded in PBS with 10 IU/ml penicillin and 10 μg/ml streptomycin to obtain 20% (w/v) tissue homogenates. Nasal swab samples and tissue homogenates were clarified by centrifugation and stored at -70°C until titration on MDCK cells. Briefly, confluent monolayers of cells were inoculated with 10-fold serial dilutions of the sample. After five days of incubation at 37°C with 5% CO_2_, virus positive MDCK cells were visualized by immunoperoxidase staining. The cells were fixed with 4% paraformaldehyde for ten minutes at room temperature and subsequently incubated with mouse anti-NP monoclonal HB-65 antibody (1:50, ATCC) for two hours. Incubation with horseradish peroxidase-conjugated goat anti-mouse polyclonal antibody (1:200, Dako) for one hour was followed by a development step with H_2_O_2_ as substrate and 3-amino-9-ethyl-carbazole (AEC) as precipitating agent. Virus titers were calculated by the method of Reed and Muench (1938) and expressed as log_10_ 50% tissue culture infective doses (TCID_50_) per 100 milligram (nasal swabs) or per gram (tissues).

### Virus neutralization assays

Virus neutralization tests were performed on MDCK cells in 96-well plates, using 100 TCID_50_ of virus per well as previously described [34]. Briefly, 2-fold serum dilutions were incubated (1h, 37°C) with 100 TCID_50_ of MDCK cell-grown virus. MDCK cells (800.000 cells per ml) were incubated with the virus-serum mixture for 24h, after which virus positive cells were visualized by immunoperoxidase staining.

### Data analysis

Because the detection limit was 1.7 log_10_ TCID_50_ per 100 milligram nasal secrete or gram tissues, samples that tested negative for virus were given a numeric value of 1.6 log_10_ TCID_50_ per 100 milligram nasal secrete or gram tissues. Samples that tested negative in the virus neutralization assay were assigned a value corresponding to half of the minimum detectable titer. For each transmission experiment, the basic reproduction ratio (R_0_) was estimated on the basis of the outcome of the experiment (final-size method) with use of the maximum likelihood estimator. Thus, the most likely reproduction ratio was estimated using the total number of animals in the experiment, the total number of susceptible and infectious animals at the beginning of the experiment, and the number of animals that became infected during the experiment. A pig was considered to be infected when it experienced seroconversion. The 95% confidence intervals (CIs) were constructed symmetrically around the estimated value of R_0_ [24].

In addition, nasal virus shedding of individual pigs was quantified by calculation of the area under the curve (AUC). The means of the AUCs were compared between different viruses using standard two–sample Mann-Whitney U tests. Differences were considered significant when p<0.05. GraphPad Prism Software, version 5, was used for statistical analyses.

### Amino acid sequences alignment

Amino acid sequences of HA proteins from H9N2 viruses isolated from swine and humans in the field were obtained from Genbank. Different human and swine amino acid sequences were compared with the HA of the A/quail/Hong Kong/G1/1997 (H9N2) strain, with special attention to amino acids at positions 190, 225, 226 and 228 because these contribute to transmission between mammals [13, 16]. Amino acid differences were identified by alignment using MEGA 6.06 software.

### RT-PCR

We compared the genetic diversity of the parental wild-type H9N2 virus and two samples from the passage four virus (which was also selected for the transmission experiments): one from the nasal mucosa (upper respiratory tract, due to the limited amount of tissue obtained the virus was amplified in MDCK cells) and another from the lungs (lower respiratory tract). The RNA from these samples was isolated using the QIAamp Viral RNA Mini Kit (Qiagen). cDNA was synthesized with the Transcriptor First Strand Synthesis kit (Roche), as described previously [35]. Two separate reactions were performed, using primers specific for the influenza A vRNAs: CommonUni12G (GCCGGAGCTCTGCAGATATCAGCGAAAGCAGG) and CommonUni12A (GCCGGAGCTCTGCAGATATCAGCAAAAGCAGG). Subsequently, all eight genomic segments were amplified in one PCR reaction using a mix containing an 1:1 mix of CommonUni12G and CommonUni12A cDNA, primer CommonUni13 (GCCGGAGCTCTGCAGATATCAGTAGAAACAAGG) (200 nM) and the Phusion High Fidelity polymerase (Thermo Scientific) [35–37]. RT-PCR was performed as described [35], with the first five PCR cycles performed with an annealing temperature of 45°C (instead of 72°C). PCR products were purified using the High Pure PCR Product Purification Kit (Roche) and elution of the DNA was in sterile ultrapure water.

### Illumina MiSeq library preparation and sequencing

150 ng of each purified RT-PCR sample was sheared with an M220 focused-ultrasonicator (Covaris) set to obtain peak fragment lengths of 400 bp. The fragment ends of 50 ng of these DNA fragments were repaired using the NEBNext Ultra DNA Library Preparation kit (New England Biolabs), followed by addition of the adaptors by using the NEBNext Multiplex Oligos for Illumina kit (Dual Index Primers Set 1, New England Biolabs). The resulting fragments were size-selected at 300 to 400 bp using Agencourt AMPure XP bead sizing (Beckman Coulter). Afterwards, indexes were added in a limited-cycle PCR (10 cycles), followed by purification on Agencourt AMpure XP beads. Fragments were analyzed on a High Sensitivity DNA Chip on the Bioanalyzer (Agilent Technologies) before loading on the sequencing chip. After the 2×250 bp MiSeq paired-end sequencing run, the data were base called and converted to Illumina FASTQ files (Phred +64 encoding).

### Next generation sequencing data analysis

Sequence data analyses were performed on the resulting Illumina FASTQ files (Phred 64+ encoding) using CLC Genomics Workbench (Version 7.0.3) following the analysis pipeline as described [35]. The processed sequencing reads were mapped to the consensus sequence obtained after *de novo* assembly of the sequencing reads for the A/quail/Hong Kong/G1/1997 (H9N2) virus stock sample, since this virus stock was used to start the virus adaptation. This consensus sequence is available through GenBank accession number (KY785896-KY785903). The raw sequencing data were submitted to the NCBI Sequence Read Archive where they can be found under project number SRP078326.

### Genetic characterization

To evaluate if the mutations described in passage four were maintained throughout the subsequent passages, the partial coding sequences of HA, M and PB1 genes were determined for all inocula from passage five onwards by direct Sanger sequencing of overlapping RT-PCR products. RNA was extracted from virus with the QIAamp Viral RNA Mini Kit (Qiagen, Valencia, CA). Reverse transcription and amplification of the genes was done by one-step RT-PCR (One-step reverse-transcription polymerase reaction kit, Qiagen, Valencia, CA) using custom specific primers (primer sequences available upon request). After amplification, RT-PCR products were purified using the Nucleo Spin Gel and PCR clean up (MACHEREY-NA-GEL GmbH&CoKG, Duren, Germany) DNA purification kit. Samples were sequenced by GATC Biotech AG (Constance, Germany) using the Sanger ABI 3730 xl platform. Sequencing was done with custom designed primers (primer sequences available upon request). HA, M and PB1 gene segments were compared at the nucleotide and amino acid (aa) level using MEGALIGN program within the DNASTAR 5.01 software package (DNASTAR, Inc., Madison, WI, USA). The complete genome sequences of A/quail/Hong Kong/G1/1997 (H9N2) were already available from GenBank (GenBank accession numbers: AF156378.1 (HA), AF156463.1 (M), AF156421.1 (PB1). The sequences generated in this study are available through GenBank accession numbers (KY785881-KY785895)

## Results

### Serial passages of avian H9N2 virus in swine is associated with a transient increase in replication

**Fig 1:**
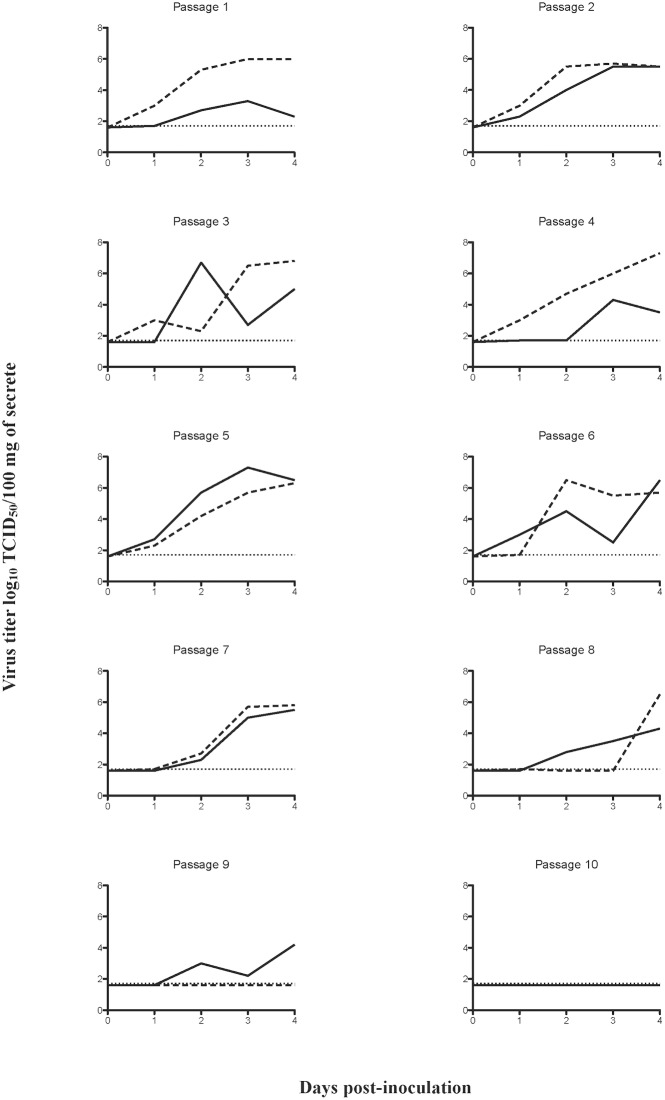
Nasal virus excretion of A/quail/Hong Kong/G1/1997 influenza virus during ten blind serial pig passages. Two pigs (solid line and dashed line) were involved in each passage, each line represents the individual virus titers in nose swabs. The detection limit of the test is indicated with a dotted line at 1.7 log_10_ TCID_50_/100 mg of secrete.

The prime goal of this study was to assess the capacity of an avian H9N2 virus to adapt to swine. For this, ten blind serial passages were performed in influenza naïve pigs. Animals were clinically scored and samples from the upper and lower respiratory tract were isolated to monitor viral loads. None of the pigs showed clinical signs. An overview of the virus titers in nasal swabs of individual pigs through the different passages is shown in Fig 1. H9N2 virus was detected in nasal swabs of all pigs, except for one pig during passage nine and both pigs during passage ten. The highest virus titers were observed during passages four and five.

On day four after inoculation, pigs were euthanized and samples from the upper and lower respiratory tract were collected to determine the viral load. Infectious H9N2 virus was found in the respiratory tract of all pigs except for those of passage ten (Table 1). The number of virus positive samples and amount of virus in the different tissue samples were in concordance with nasal virus excretion results, with considerable variation between individual pigs and passages (compare Table 1 and Fig 1). Interestingly, replication rates were higher in the upper respiratory tract (nasal mucosa: respiratory and olfactory parts) with 36 out of 40 (90%) samples testing positive, than in the lower respiratory tract (trachea and lungs) with 65 out of 134 (49%) samples positive. In the upper respiratory tract, virus was isolated at similar frequency from the olfactory and the respiratory parts of the nasal mucosa. After the first three passages virus was isolated from some of the lower respiratory tract samples, indicating that at early passages the virus did not replicate uniformly in the entire lung. Only during passage five, after inoculation with passage four virus, infectious virus was clearly detected in all samples, suggesting replication in the entire respiratory tract. Remarkably, following passage five, the virus seemed to gradually have lost its ability to replicate in the lower respiratory tract and eventually, at passage 10, infectivity was completely lost.

**Table 1:** Distribution of A/quail/Hong Kong/G1/1997 avian influenza virus in the respiratory tract during ten serial passages in pigs.

Number of passage	Pig number	Virus titer (log_10_ TCID_50_/gram of tissue) at day 4 post-inoculation^a^								
		Nasal mucosa respiratory part	Nasal mucosa olfactory part	Proximal trachea	Distal trachea	Apical+cardiac lobe right	Apical+cardiac lobe left	Diaphragmatic lobe right	Diaphragmatic lobe left	Accessory lobe
1	#1	4	3	2^b^		4.7	<^c^	4.5	<	<
	#2	5.2	6.5	2.5		4.5	<	<	<	1.7
2	#3	5.5	4.5	2		<	<	<	<	<
	#4	6.5	4.2	<		<	<	<	<	2
3	#5	6.5	6.5	<		2	<	<	<	<
	#6	5	5.3	1.7		<	<	<	1.7	1.7
4	#7	7.2	6.2	<	<	<	1.7	<	1.7	<
	#8	5.7	3.3	4.5	4.2	5	1.7	1.7	<	4.3
5	#9	5	5.5	3.3	4.3	5.3	5.2	3.7	5.2	2.3
	#10	7	7.7	5.8	5.3	3.8	2.2	2	5	4.7
6	#11	4.7	5.5	3.7	2.7	4.5	2.6	<	<	5.7
	#12	5.7	4.5	3.5	5.3	5.5	2	4.4	4.4	5.5
7	#13	7	2.8	5.5	<	<	<	<	<	1.7
	#14	5.3	4	2.7	5	6.4	4.7	4.5	3.8	5.7
8	#15	3.7	5	<	<	<	2.3	<	<	<
	#16	5.3	1.7	3.5	5.5	2	<	4.5	3.3	4.7
9	#17	2	1.7	<	<	<	<	<	<	1.7
	#18	4.2	2.3	<	<	<	<	<	<	2.3
10	#19	<	<	<	<	<	<	<	<	<
	#20	<	<	<	<	<	<	<	<	<

^a^ Virus titers are shown for each individual pig (#).

^b^ Proximal and distal trachea samples were combined during passages one to three.

^c^ < detection limit (1.7 log_10_ TCID_50_/gram of tissue).

### H9N2 virus becomes contact transmissible after four serial passages in pigs

To assess the level of adaptation of the virus after serial blind passages in pigs, three different viruses were tested in three independent direct contact transmission experiments. As a positive control, we used the A/California/04/2009 (A/Cal/04/09) pH1N1 virus, which is representative of swine-adapted influenza viruses [38–40]. The parental A/quail/Hong Kong/G1/1997 (A/Qa/HK/P0) H9N2 virus served as starting material for the serial passages. The passage four H9N2 virus sample (A/Qa/HK/P4) that was used to inoculate animals in passage five was also selected for the transmission experiment, because based on virus titers in nasal swabs and respiratory tract samples this virus seemed to have gained the highest swine-adaptation. All three viruses were amplified in MDCK cells before inoculation of the index pigs and the transmission experiment was performed in triplicate for each virus.

**Fig 2:**
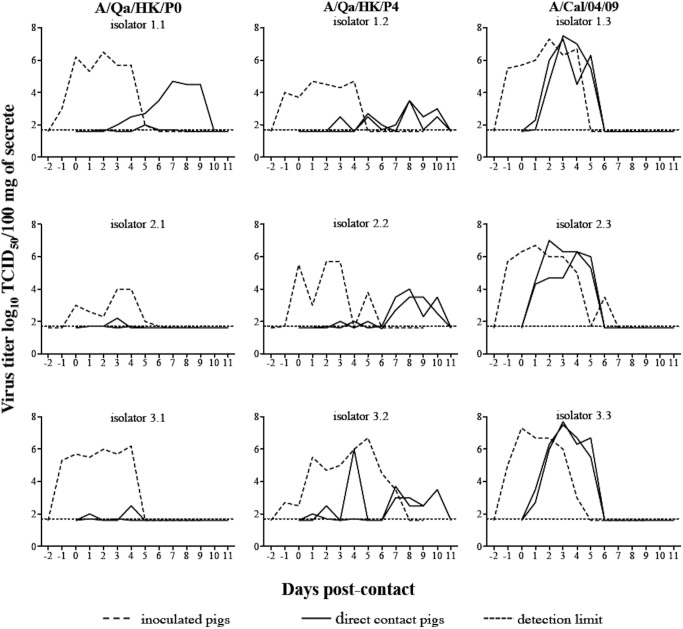
Nasal virus excretion and direct contact transmission of A/Qa/HK/P0, A/Qa/HK/P4 and A/Cal/04/09 influenza viruses in pigs. Three pigs were intranassally inoculated with 6.5 log_10_ TCID_50_ of the indicated virus per pig and individually housed in different isolators. Forty-eight hours later, two direct contact animals were co-housed with the inoculated pigs. Each graphic is identified with a two digits number: the first one corresponds to the isolator number and the second to the virus tested. Therefore, each column represents a different virus and each row represents a different isolator. The detection limit (dotted line) of the test was 1.7 log_10_ TCID_50_/100 mg of secrete.

Fig 2 shows the nasal virus shedding of the inoculated and the co-housed direct contact animals. All piglets remained clinically healthy, based on clinical observation scores. All inoculated animals excreted virus between days one and eight post-inoculation. Comparison of the AUC revealed lower virus excretion for A/Qa/HK/P0 (18.4) and A/Qa/HK/P4 (19.1) than for A/Cal/04/09 (26.6), although these differences were not statistically significant (p<0.05). Of all nine inoculated animals five reached a maximum virus titer of ≥ 6.5 log_10_ TCID_50_/100 mg of secrete. All inoculated animals developed antibodies against the homologous virus (Table 2). Higher antibody titers were observed in A/Cal/04/09 inoculated pigs than in H9N2 infected animals.

**Table 2:** Antibody responses in inoculated and direct contact pigs involved in transmission experiments measured by VN test.

	Number of antibody positive pigs (range of antibody titers)							
	Inoculated pigs (n = 3)				Direct contact pigs (n = 6)			
Virus strain	0	16	23	30 dpi^a^	0	14	21	28 dpc^b^
A/Qa/HK/P0	0 (<2)	3 (2–12)	3 (6–24)	3 (6–12)	0 (<2)	2 (24–32)	2 (8–16)	2 (16–24)
A/Qa/HK/P4	0 (<2)	3 (32–384)	3 (64–128)	3 (48–128)	0 (<2)	1 (4)	5 (2–64)	5 (2–12)
A/Cal/04/09	0 (<2)	3 (128–192)	3 (192–384)	3 (512–768)	0 (<2)	6 (192–1024)	6 (256–1536)	6 (192–1024)

^a^ dpi: days post-inoculation.

^b^ dpc: days post-contact.

Only one out of six direct contact animals in the A/Qa/HK/P0 group shed virus during more than five days resulting in a mean AUC of 2.8. In contrast, all six animals that were co-housed with an A/Qa/HK/P4 virus inoculated pig, excreted virus for five days or more. The virus excretion was not homogeneous over time (mean AUC of 5.9) and peak viral loads in contact animals were approximately 100-fold lower compared to the viral titers in the nasal excretions of the A/Qa/HK/P4 index pigs (Fig 2). In contrast, all direct contact pigs in the A/Cal/04/09 group shed virus in a pattern that is very similar to that of the inoculated pigs, with a mean AUC of 19.7, and similar peak viral titers. The difference between the AUC values of the A/Qa/HK/P4 direct contact pigs and the A/Qa/HK/P0 direct contact pigs was not statistically significant (p<0.05). However, both groups shed significantly less virus than A/Cal/04/09 contact pigs (p<0.05). Five out of six A/Qa/HK/P4 contact animals had antibodies against the homologous virus, while only two animals that had been exposed to A/Qa/HK/P0 infected index animals seroconverted. As expected, all six A/Cal/04/09 contact animals seroconverted, with higher titers than the ones detected in the H9N2 direct contact animals. These data allowed us to determine the R_0_ value, a measure for the transmissibility of an infectious agent. R_0_ was 0.76 (95% CI: 0.10–5.49) for A/Qa/HK/P0 and 2.27 (95% CI: 0.54–9.29) for A/Qa/HK/P4. Because all A/Cal/04/09 contact pigs became infected, the estimated R_0_ value was ∞ (95% CI: 0.83-∞) for this experimental setting. In summary, A/Qa/HK/P4 showed enhanced transmission compared to A/Qa/HK/P0, but both viruses transmitted less efficiency than A/Cal/04/09.

**Fig 3:**
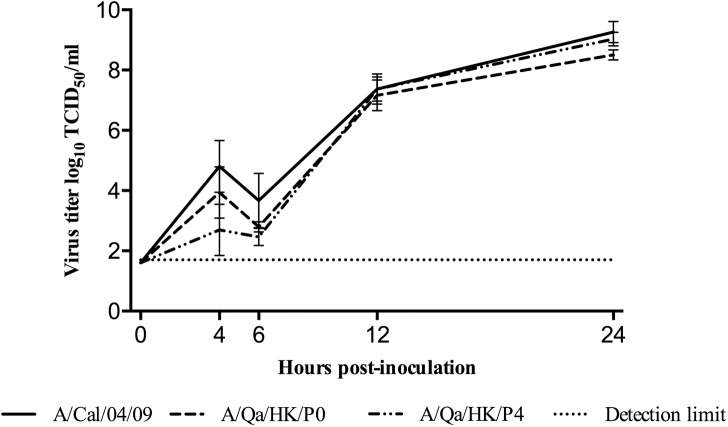
The effect of the serial passaging on the amount of virus produced over the course of the experiment using a single-step growth assay in MDCK cells. Each data point on the curve is the mean ± SD of the three independent experiments.

In addition, we evaluated the replication kinetics of the viruses tested in the transmission experiments by determining the single-step growth curve in MDCK cells for each virus. As demonstrated in Fig 3 the growth kinetics of the three viruses were very similar. Therefore, the results show that the enhanced *in vivo* transmission of the A/Qa/HK/P4 virus did not have an impact on the *in vitro* replication kinetics of the virus in a continuous cell line.

### Mutations associated with pig adaptation

We compared the viral diversity present in the H9N2 virus stock that was used to start the serial passages with the viral diversity present in the virus sampled after passage four from the nasal mucosa (after a single round of amplification on MDCK cells) and the lung. The viral diversity was analysed in virus (pooled from two pigs) sampled after passage four, since higher virus shedding and higher viral replication in the upper and lower respiratory tract was obtained upon inoculation with this virus. It was anticipated that a comparison of the viral diversity present in the virus from the upper and lower respiratory tract would enable us to determine if these two airway compartments favour the acquirement of a different set of mutations for optimal adaptation. In addition, sequencing of the virus stock is needed to determine if the detected pig mutations were already present in the virus inoculum at the start of the serial passages or if they arose *de novo*. Illumina MiSeq deep sequencing was performed on full genome RT-PCR products of the samples. Nucleotide variants that differ from the consensus sequence of the A/quail/Hong Kong/G1/1997 (H9N2) virus stock sample that was used to start the serial passages and that occur at a frequency of 5% or more are shown in Table 3. Two mutations were present in the starting material. These are non-synonymous mutations that alter two adjacent amino acids of the polymerase acidic protein (PA). Interestingly, after four passages a substitution of aspartic acid by glycine at position 225 of the HA RBS (HA-D225G) was present in 80.1% of the sequences derived from the viral population of the nasal mucosa and in 99.6% of the viral population of the lung tissue sample. Another substitution was close to 100% present in lung homogenate virus, resulting in an alanine to threonine substitution at position 29 (M2-A29T) in matrix 2 protein (M2). In addition, two more substitutions appeared in the nasal mucosa after four passages: one in PA and another in non-structural 1 protein (NS1). These two mutations were present at a frequency below 10%. In the lung virus samples, three mutations newly appeared with frequencies ranging from 23.37 to 44.19%. These mutations were present in basic polymerase 1 (PB1), HA and nucleoprotein (NP).

**Table 3:** Mutations present in parental and passage four H9N2 virus isolated from the nasal respiratory mucosa or from the lung.

Segment	Nucleotide position	Reference	Mutation	Amino acid change	Frequency A/Qa/HK/P0	Frequency A/Qa/HK/P4 (nasal mucosa)	Frequency A/Qa/HK/P4 (lung)
PB1	559	A	G	Glu172Gly	<^a^	<	42.34
PA	1239	G	A	Glu399Lys	<	5.00	<
	1506	A	G	Lys488Glu	10.25	11.21	7.84
	1509	T	A	Cys489Ser	21.37	21.79	10.27
HA	207	A	G	His44Arg	<	<	23.37
	750	A	G	Asp225Gly	<	80.88	99.96
	959	T	C	Phe295Leu	<	<	44.19
NP	1347	G	A	Ala428Thr	<	7.29	10.99
M	818	G	A	M2: Ala29Thr	<	23.26	98.33
NS	191	A	G	NS1: Thr49Ala	<	9.58	<

The viral gene segment is indicated, along with the nucleotide position and substitutions, the predicted amino acid change and its position and finally the frequency (percentage) of sequence reads with the detected mutations. Only nucleotide substitutions that resulted in amino acid changes and appeared at a frequency ≥ 5% in the reads are shown. The virus in the nasal mucosa sample was amplified on MDCK cells before sequencing.

^a^ <: mutation not detected using 0.5% as variant calling threshold

To evaluate if the substitutions present at the highest frequency in the passage four virus (HA-D225G, HA-F295L, M2-A29T and PB1-E172G) were maintained during the subsequent passages we sequenced the HA, M and PB1 coding-genes of the virus present in the inocula used to infect pigs from passages six to ten. Interestingly, the substitutions observed in the A/Qa/HK/P4 nasal mucosa were maintained during all passages. However, the substitutions detected in the lung sample were no longer found in further passages.

## Discussion

Avian H9N2 viruses play a pivotal role in the ecology of influenza in poultry in Eurasian countries. Since the late 1990’s, recurrent dead-end infections with H9N2 viruses have been reported in pigs and humans [6, 41, 42]. Moreover, serological studies in Asian poultry workers and in Chinese pigs revealed significant exposure to H9N2 [30, 43, 44]. The continuous exposure to avian H9N2 viruses might pose a real threat for public health if these viruses would acquire mutations that allow them to transmit efficiently between mammals. Pigs are considered intermediate hosts in which avian H9N2 influenza viruses may acquire such mutations. We have therefore examined whether blind serial passages of an avian H9N2 virus in swine would result in swine-adaptive mutations. We consider an influenza virus as swine-adapted if it succeeds to transmit between pigs in an experimental setting with a similar efficiency as endemic swine influenza viruses. Therefore, we also performed a transmission experiment with the parental and at least partially swine adapted H9N2 virus.

Our results confirm the susceptibility of pigs to intranasal inoculation with avian H9N2 influenza viruses reported in previous experimental studies and in nature [1, 25, 29, 30]. However, they are not in agreement with two previous studies in which avian H9N2 viruses failed to replicate in pigs. De Vleeschauwer *et al*. (2009) inoculated pigs intranasally with A/chicken/Belgium/818/1979 (H9N2) and found that this strain was not excreted at all by the inoculated pigs [24]. Though genetic information of this specific isolate is not available, the lack of replication in the inoculated pigs was likely due to the fact that European H9N2 isolates belong to the Y439-like lineage, which likely lack the ability to replicate in pigs [3, 30]. In the second study, Qiao *et al*. (2012) used the same A/quail/Hong Kong/G1/1997 (H9N2) strain to inoculate pigs intratracheally and they detected nasal virus excretion in only one out of twelve inoculated animals, albeit at low virus titers [28]. The very poor capacity to replicate in swine compared to our findings with A/quail/Hong Kong/G1/1997 (H9N2) can be explained by the different inoculation routes used in our and Qiao’s study, which will result in different sites of primary virus replication. Indeed, after intratracheal exposure replication in the nasal mucosa and nasal virus excretion is secondary to and dependent on replication in the lower airways. Thus, intratracheal inoculation thus usually results in delayed nasal virus excretion and lower nasal virus titers as compared to the intranasal inoculation [45]. Moreover, we confirmed previous reports describing the susceptibility of pigs to AIVs of different subtypes [24, 25, 46]. The parental H9N2 virus replicated in the upper respiratory tract at low to moderate titers while most samples of the lower respiratory tract tested virus negative. The relative lack of Siaα2,3Gal receptors in human and swine upper respiratory tract is thought to restrict the efficient replication of AIVs in mammals. Therefore, a shift from Siaα2,3Gal to Siaα2,6Gal receptor-binding preference has been appointed as a critical step in the adaptation of AIVs to mammalian hosts [47]. For H9, as well as for H3, H5 and H7 avian subtypes, the HA-Q226L substitution contributes to increased Siaα2,6Gal receptor preference and is associated with higher replication rates and transmissibility in pigs and ferrets [21, 23, 32, 46, 48]. Though our parental H9N2 virus already contains the HA-Q226L substitution, the virus distribution and replication rates described in our study were similar to those observed in pigs that had been inoculated with H5N2 AIV subtype virus with a “pure” avian genome, i.e. with HA-226Q [45]. Our data therefore question the role of the HA-Q226L substitution in replication efficiency in pigs but are in line with more recent studies, which demonstrate that residue 226 in HA is not strictly determinative for the replication of H9N2 viruses in pigs or ferrets [29]. Comparative studies with HA-226L and HA-226Q would be required to evaluate the effect of the HA-Q226L substitution in pigs. In contrast to the parental H9N2 virus, the virus isolated after four pig passages replicated homogeneously throughout the entire respiratory tract. We compared pig-to-pig transmission of this virus with that of the parental H9N2 and pH1N1. While the parental H9N2 virus largely failed to transmit to direct contact pigs, the fourth passage virus showed enhanced transmissibility as demonstrated by the AUC and the R_0_. This virus showed also higher transmission efficiency when compared with previous studies with H9N2 as well as other avian influenza virus subtypes in pigs. In all of these studies the direct contact pigs excreted no or minimal amount of virus [24, 29, 30]. However, transmission of the fourth passage virus remained far less efficient than that of endemic swine influenza viruses. Although the three viruses showed different phenotypes in pigs, their growth kinetics in MDCK cells were similar, indicating a poor correlation between the *in vivo* phenotype and *in vitro* replication rates on continuous cell lines. Furthermore, from passage seven onwards the virus replication progressively decreased until it was lost. This result may be explained by the generation of a narrow bottleneck after passage four due to the strong genetic pressure posed by the experimental set up, which may decrease the diversity of the viral population which can hamper the adaptation process [49, 50].

**Fig 4:**
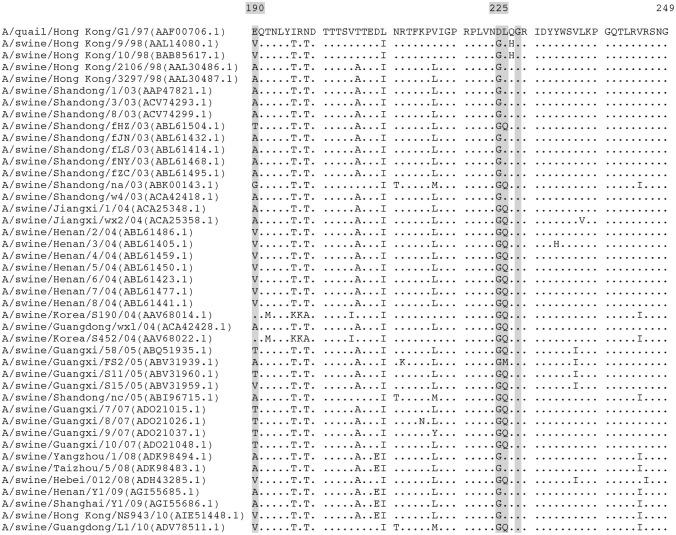
Alignment of the deduced amino acid sequences in the HA of all swine H9N2 isolates (n = 47) available in Genbank on 22^nd^ August 2016. The name of each strain included in the analysis is followed by the accession number between brackets. Residues at positions 190, 225, 226 and 228 are highlighted in gray. Amino acids that are different from those in A/quail/Hong Kong/G1/1997 are shown, conserved residues are shown as dots.

**Fig 5:**
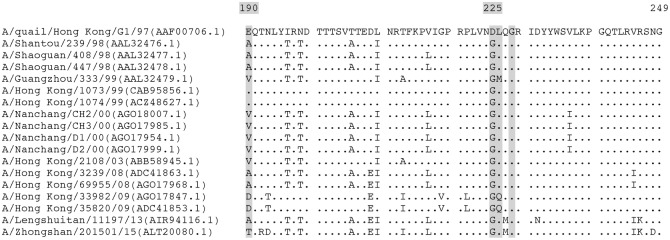
Alignment of deduced amino acid sequences in the HA of all human H9N2 isolates (n = 12) available in Genbank on 22^nd^ August 2016. Strain names are followed by accession numbers (between brackets). Residues at positions 190, 225, 226 and 228 are highlighted in gray. Amino acids that are different from those in A/quail/Hong Kong/G1/1997 are shown, conserved residues are shown as dots.

Genetic changes during AIV passage in animals are not predictable [51]. To better understand the effect of the serial passaging in the virus population and the behaviour of the virus in our transmission experiment we compared the viral diversity in the original wild-type H9N2 virus with the virus isolated from nasal mucosa and lung after four passages. We observed that the upper and lower respiratory tract selected for different virus variants. Moreover, the mutations identified in the upper respiratory tract were maintained during the subsequent passages, while those found in the lung virus samples were not maintained in the subsequent passages. Although more genetic analyses might be necessary to better understand this discrepancy between the viral selection pressure in the upper and lower respiratory tract, this points to a possible role of the mutations detected in the lung samples in the enhanced replication and transmissibility. Only one out of the ten mutations we found, the HA-D225G substitution, has been previously described in the literature [16, 52, 53]. This mutation arose *de novo* in both the upper and lower respiratory tract after four passages and it was associated with a higher replication efficiency during passage five. Interestingly, this mutation was also present in all forty-seven swine and twelve human H9N2 field isolates available in Genbank (see Figs 4 and 5). In 1918 and 2009 H1N1 pandemic viruses, HA-D225G substitution has been associated with increased Siaα2,3Gal tropism conferring those viruses dual Siaα2,6Gal and Siaα2,3Gal receptor binding affinity [54–56]. In pigs, as well as in humans, Siaα2,3Gal receptors are predominantly found in the lungs and not in the upper respiratory tract [14]. The emergence of this mutation after four passages could therefore explain the enhanced replication in the lungs. Nevertheless, in *in vitro* sialylglycoprotein binding assays H9N2 viruses containing HA-225G and HA-226L, as our fourth passage virus, showed only slightly increased Siaα2,6Gal binding affinity [20, 54]. Though specific binding tests would be needed to clarify the exact role of the HA-D225G substitution, our data suggest that it is an important marker for adaptation of H9N2 AIVs to pigs.

In summary, our results do not reject the theory of the pig as an intermediate host for H9N2 AIVs, but demonstrate that adaptation of these viruses to pigs is a complex process and that the mutations selected after four passages in pigs were not sufficient to confer efficient pig-to-pig transmission. It also shows that additional molecular features may be required for efficient transmission of avian H9N2 viruses in pigs. Qiao *et al*. (2012) and Obadan *et al*. showed that reassortant H9N2 viruses containing pH1N1 internal genes improved replication and transmissibility in pigs, although they were still less efficient than pH1N1 virus. In previous H9N2 adaptation studies in mammals, efficient transmission was only reached with ferret-passaged reassortant H9N2 viruses containing either human seasonal H3N2 or pH1N1 internal genes [31, 57]. Therefore, the combination of mammalian adapted internal genes with serial passaging may be required to select a fully transmissible H9N2 virus.
